# Zinc as diagnostic marker of cancers

**DOI:** 10.1186/1897-4287-13-S2-A4

**Published:** 2015-11-26

**Authors:** Katarzyna Kaczmarek, Magdalena Muszyńska, Katarzyna Jaworska-Bieniek, Wojciech Marciniak, Grzegorz Sukiennicki, Marcin Lener, Katarzyna Durda, Tomasz Gromowski, Tomasz Huzarski, Tomasz Byrski, Jacek Gronwald, Oleg Oszurek, Cezary Cybulski, Tadeusz Dębniak, Antoni Morawski, Anna Jakubowska, Jan Lubiński

**Affiliations:** 1 Department of Genetics and Pathology, International Hereditary Cancer Center, Pomeranian Medical University, Szczecin, Poland; 2 Read - Gene, S.A., Grzepnica, Poland

## Background

Zinc is a micronutrient which is essential for human health, playing a role as a cofactor of enzymes such as dehydrogenases, peptidases and component of zinc finger domains. In organism zinc is involved in metabolic pathways, immune processes, maintaining ion balance between other elements. Recently, it has been reported that zinc may play role in chemoprevention, and its level may be associated with occurrence of cancers.

## Aim of the study

The aim of the study was to evaluate a possible correlation between serum Zn level and occurrence of prostate, colorectal and laryngeal cancers. This will allow to determine whether serum Zn level may be used as an indicator which patients should be subjected for further cancer diagnostics.

## Material and methods

The study has been conducted in 3 groups: 197 prostate cancer cases and 197 controls, 101 colorectal cancer cases and 101 controls, 109 laryngeal cancer cases and 109 controls.

Zinc level in serum was measured in all individuals by inductively coupled plasma mass spectrometry (ICP-MS) using Elan DRC-e ICP-Mass Spectrometer, Perkin Elmer.

After obtaining results from mass spectrometry, individuals in each group were divided into 4 quartiles. Comparison of number of cases and controls was performed in each quartile. Risk of cancer occurrence was evaluated with regards to the reference category - the lowest zinc level.

## Results

We have observed that high serum Zn level (>991 µg/l) was associated with higher incidence of prostate cancers (OR = 2.43; p < 0.003; CI 1.370 - 4.309). In contrast, high level of Zn (>959 µg/l) was associated with decreased incidence of laryngeal cancers (OR = 0.08; p < 0.0001; CI 0.03122 - 0.1918). In group of colorectal cancers we have not found any correlation between Zn level and cancer occurrence.

## Conclusions

Results of our study suggest that high level of Zn may be an indicator for prostate examination (ex. PSA, biopsy) and low level of Zn may be an indicator for laryngeal examination. Serum Zn level assessment may improve cancer screening by suggesting which patients should be subjected for further testing. Such procedure may increase early detection of cancer. Our results seem to be promising and the study will be also conducted in groups of breast and lung cancers.

## Acknowledgements

This work was financially supported by National Science Centre grant no. 2012/07/N/NZ4/02433.

